# Imaging evaluation of continuous extrapleural intercostal nerve block for minimally invasive cardiac surgery: a case report

**DOI:** 10.1186/s40981-021-00450-y

**Published:** 2021-06-09

**Authors:** Misa Terauchi, Hiroai Okutani, Daisuke Ishimoto, Noriko Shimode, Yumiko Takao, Munetaka Hirose

**Affiliations:** Department of Anesthesiology and Pain Medicine, Hyogo College of Medicine, 1-1, Mukogawa-cho, Nishinomiya, Hyogo 6638501 Japan

**Keywords:** Continuous extrapleural intercostal nerve block, Minimally invasive cardiac surgery, Postoperative pain, Contrast medium, Intercostal thoracotomy

## Abstract

**Background:**

Spinal nerve block is difficult with minimally invasive cardiac surgery (MICS), because of the risk of serious bleeding complications due to full heparinization. Continuous extrapleural intercostal nerve block (CEINB) is a postoperative pain treatment for intercostal thoracotomy, with fewer complications. Here, we report a case in which imaging evaluation of CEINB with contrast medium was conducted to anatomically confirm the spread of local anesthetics after MICS.

**Case presentation:**

A 65-year-old woman with severe mitral regurgitation underwent mitral valve plasty under general anesthesia via right-sided mini-thoracotomy. A CEINB catheter was placed before the incision was closed, without creating a conventional extrapleural pocket. We conducted an imaging evaluation with a contrast medium via the inserted catheter and confirmed sufficient spread around the intercostal nerve area. In addition, postoperative pain was well controlled by the nerve block.

**Conclusions:**

Imaging evaluation of CEINB with contrast medium could increase analgesic quality and decrease complications post-MICS.

## Background

Although minimally invasive cardiac surgery (MICS) is a feasible alternative to conventional full sternotomy with a low incidence of transfusion and general morbidity [1], intercostal thoracotomy causes severe prolonged postoperative pain, which is closely associated with postoperative pulmonary complications. Intercostal nerve block effectively controls pain after MICS; however, repeated administration of local anesthetics is required for adequate analgesia. Continuous infusion of local anesthetics via an intraoperatively placed catheter provides good analgesia after MICS [2]. Continuous extrapleural intercostal nerve block (CEINB) is a postoperative pain treatment for thoracotomy with fewer complications; a catheter is placed into the extrapleural intercostal nerve area from the end of the thoracotomy incision [3]. Here, we present the case of a patient who underwent CEINB without the creation of an extrapleural pocket, to relieve postoperative pain after MICS. Imaging using a contrast agent confirmed the extent of local anesthetic spread.

## Case presentation

A 65-year-old woman (height, 161 cm; weight, 46 kg) with severe mitral regurgitation was scheduled for MICS with mitral valve plasty via right-sided mini-thoracotomy, under general anesthesia. Although mitral regurgitation had been diagnosed in childhood, she noticed exertional dyspnea at 64 years of age. She had no other medical history and took no medications preoperatively. We explained the CEINB procedure and imaging evaluation in detail and obtained written informed consent.

**Fig. 1:**
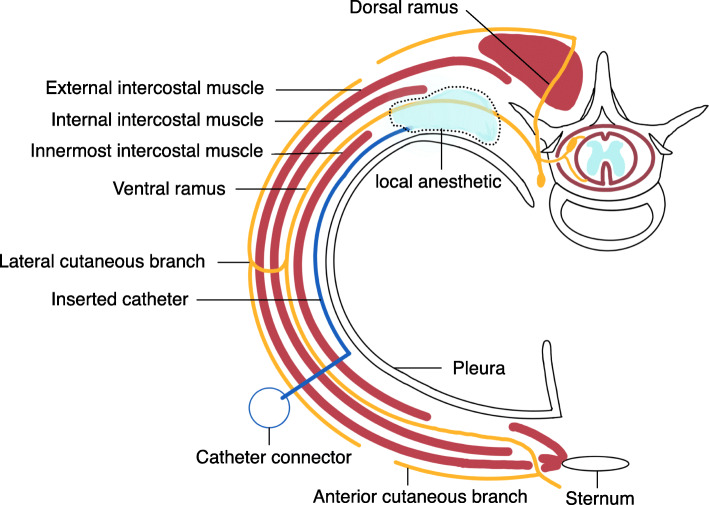
Schematic drawing of the continuous modified intercostal nerve block and the spread of solution. The catheter was inserted from the outer edge of the incision and placed between the innermost intercostal muscle and parietal pleura

On arrival in the operating room, she had stable hemodynamic parameters. We initiated preoperative monitoring including standard monitoring, electroencephalography using the bispectral index monitor and cerebral/somatic oximetry using INVOS™ (Medtronic, Dublin, Ireland), and CCO/SvO_2_ monitoring using Vigilance® (Edwards Lifesciences, Irvine, CA, USA). General anesthesia was induced with 200 μg fentanyl and 4 mg midazolam after pre-oxygenation with 100% oxygen. We administered 50 mg rocuronium after induction, and a 35-Fr left double-lumen endotracheal tube was carefully inserted without complications. Anesthesia was maintained with oxygen, air, sevoflurane (1–1.5%), remifentanil (0.1–0.35 μg/kg/min), and intermittent administration of rocuronium and fentanyl. Invasive monitoring included a radial arterial line and Swan-Ganz catheter; a 7.5-Fr vascular access sheath was inserted through the right internal jugular vein. In the supine position with 30° elevation of the right side, she underwent a MICS mitral procedure through a right thoracotomy approach via a 5.5-cm incision at the fourth intercostal space. Anesthesia was maintained with propofol (4–6 mg/kg) during cardiopulmonary bypass. Following administration of intravenous unfractionated heparin (18,000 units), femoral-femoral CPB was established, and mitral valve plasty was performed. In total, 500 μg of fentanyl was administered during the operation: 300 μg before the start of cardiopulmonary bypass and 200 μg after withdrawal of CPB. The surgeon placed the catheter for CEINB in the following steps before closing the chest (Fig. 1).

First, a syringe filled with isotonic saline was connected to a long 8-Fr vascular access sheath (8Fr × 25 cm, Radifocus® introducer IIH, Terumo Corporation, Japan), which was inserted from the outer edge of the incision to between the innermost intercostal muscle and parietal pleura. To avoid damaging the parietal pleura and blood vessels, the blunt catheter with a soft tip was used instead of a needle. The placement was confirmed by injecting saline and observing the elevation of the parietal pleura. When the tip of the sheath reached the posterior thorax, 20 mL of 0.25% levobupivacaine was administered. The tip of a catheter (19-Ga × 90 cm, Arrow®FlexTip Plus® Epidural Catheter, Teleflex Medical Japan, Japan) was then placed into the posterior thorax via the sheath. The surgery proceeded uneventfully, and the vital signs remained stable.

**Fig. 2:**
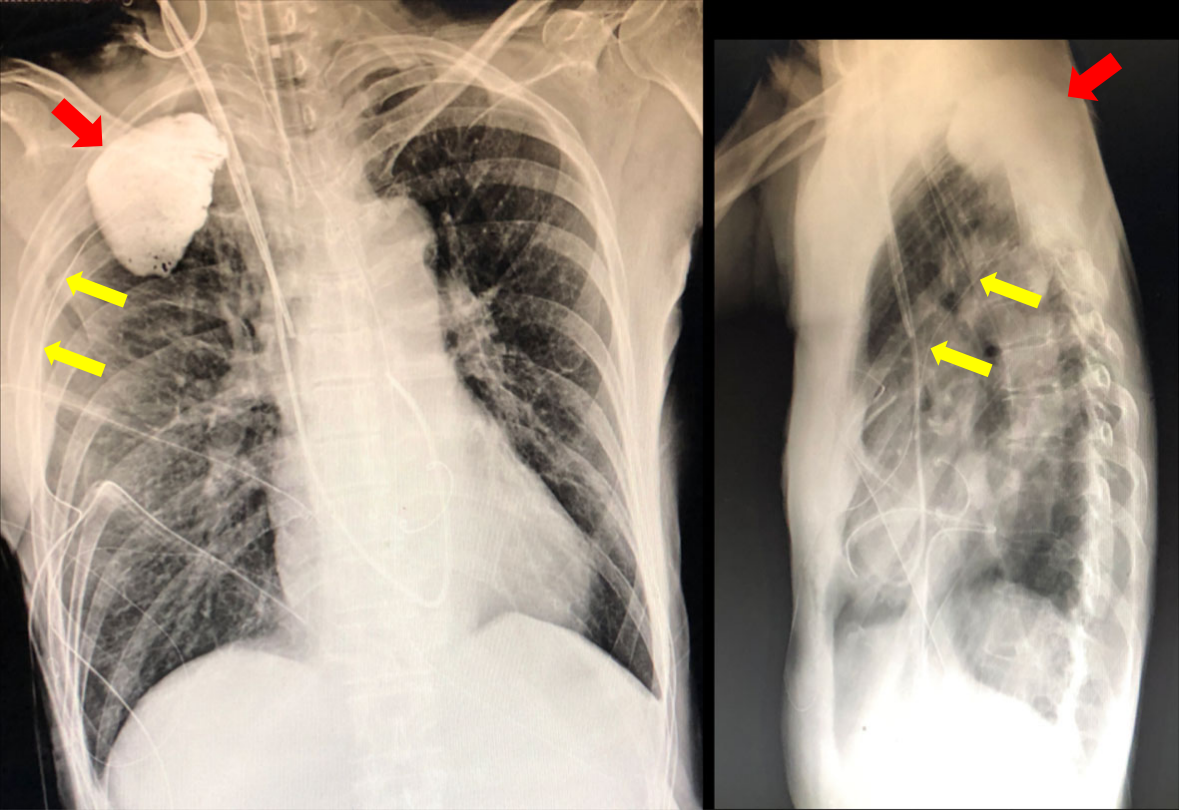
Frontal and lateral chest radiograph images showing the spread of contrast medium. The contrast medium was localized across the 3rd to the 5th intercostal area in the posterior chest. Yellow arrows indicate the inserted catheter, and red arrows indicate the administered contrast medium

After surgery, we assessed the general condition by plain chest radiography and simultaneously determined the appropriate positioning of the catheter tip; 20 mL of iohexol (Omnipaque 240®, GE Healthcare, Japan) was then injected through the catheter to confirm the spread of the local anesthetic and the catheter position. The contrast medium was distributed between the parietal pleura and innermost intercostal muscle across the 3rd to the 5th intercostal areas (Fig. 2); 0.17% levobupivacaine was then continuously administered at 4 mL/h using a disposable continuous infuser (Bezelfuser, TORAY Medical, Japan). In addition, a patient-controlled analgesia apparatus was provided at 3 mL for postoperative pain, with a lock-out time of 30 min. The patient was moved to an intensive care unit and extubated 4 h after transfer.

Pain intensity was assessed by ICU nurses using a numeric rating scale ranging from 0 to 10. During extubation, the patient rated 0/10 for postoperative resting pain and 4/10 for movement pain without other analgesics. She was discharged to the general ward on the day after surgery; patient-controlled analgesia was activated only once on the second postoperative day. Both resting pain and movement pain were subsequently rated as 0/10; administration of local anesthetics was continued. She did not receive any other analgesics, and the analgesic catheter was removed 72 h after surgery. The patient was discharged on postoperative day 12 without any complications.

## Discussion

Intercostal thoracotomy is one of the most painful surgeries; various methods have therefore been considered to reduce associated postoperative pain. The most common management consists of epidural anesthesia and systemic narcotics [4]; however, regional anesthesia is often administered by anesthesiologists. In particular, the paravertebral block is usually performed for intercostal thoracotomy [5]. However, as CPB requires anticoagulants, the risk of hematoma is a major concern in spinal nerve block [6]. Erector spinae plane block (ESPB) and retro laminar block (RLB) have been suggested as safe, avoiding the risk of perimedullary hematoma due to full heparinization [7, 8]. However, the effectiveness of a single dose is limited, and even continuous administration via ESPB or RLB may not be sufficient for the spread of local anesthetics [9]. A previous report has shown that ESPB has a broader analgesic effect than RLB; however, with ESPB, it is difficult to sufficiently infiltrate the intercostal nerve at the ventral ramus [10].

Sabanathan et al. described an intercostal nerve block technique, in which a local anesthetic is continuously injected using an intraoperatively placed indwelling extrapleural catheter [2]. Although CEINB seems to be less popular due to inferior pain management compared to that of other nerve blocks, it offers three advantages: it is quick, safe, and has a stable effect. First, surgeons can perform the operation quickly without ultrasound equipment or posture change, shortening operation times. The present CEINB method utilizes a small incision similar to MICS, as surgeons do not need to create the pleural pocket. Second, as surgeons can directly visualize catheter insertion at the extrapleural area, the approach is safe; the incidence of adverse events is likely to be low. However, pleural injury and aberrant insertion into the blood vessels can lead to serious complications; great care should therefore be taken. Anatomically, the innermost intercostal muscle becomes thinner at the dorsal intercostal space and moves to the innermost intercostal space; thus, the surgeon should exercise caution not to damage the parietal pleura when inserting the sheath catheter. In addition, the catheter must be carefully inserted to avoid aberrant placement or injury of the intercostal vessels. Therefore, we planned imaging evaluation to detect the spread of local anesthetics and any complications, especially aberrant insertion into the blood vessels. Anatomically, the spinal nerve immediately divides into dorsal and ventral rami after exiting the intervertebral foramen. The ventral ramus continues anterolaterally and becomes the intercostal nerve; it then enters between the internal and innermost intercostal muscles (Fig. 1). In this case, the contrast medium was localized across the three adjacent intercostal levels before entering between the internal and innermost intercostal muscles; the analgesic effect of the local anesthetic was sufficient, and the patient had no complications. Third, unlike epidural analgesia and PVB, this technique also serves to maintain stable vital signs, as the confirmed spread is unlikely to affect sympathetic nerves; however, this implies that it does not adequately block the sympathetic trunk, which mediates pain signals from visceral sympathetic afferents. The advantage of this confirmation is that it only requires the use of a contrast medium and can be easily performed during routine postoperative chest radiography. Moreover, confirmation by contrast medium provides excellent detection of aberrant insertion into the blood vessels and pleural injury. In addition, the catheter tip can be adjusted by withdrawal after surgery, if the findings on the administration of contrast medium suggest any abnormality. However, it needs to be avoided in patients with renal failure or allergy to contrast medium; in these cases, the use of ultrasound or dye solution evaluation should be considered.

Postoperative pain may be affected by both the intercostal incision site and intercostal nerve compression by the rib retractor and intercostal and pectoralis muscle dissection; thus, nerve block should be performed immediately after thoracotomy. It may effectively prevent post-thoracotomy pain syndrome. Numerous detailed prospective studies on MICS with contrast medium are needed for comparison of CEINB, paravertebral block, ESPB, and RLB from safety and analgesia perspectives.

In conclusion, we confirmed that the contrast medium was sufficiently distributed around the intercostal nerve via the CEINB catheter. Moreover, CEINB achieved satisfactory pain management for MICS with local anesthetics alone, without complications. Imaging evaluation of CEINB with contrast medium could be useful for increasing analgesic quality and reducing post-MICS complications.

## Data Availability

Not applicable

## Abbreviations

MICS: Minimally invasive cardiac surgery
CEINB: Continuous extrapleural intercostal nerve block
PCA: Patient controlled analgesia
ESPB: Erector spinae plane block
RLB: Retro laminar block

## References

[1] Greelish JP, Cohn LH, Leacche M, Mitchell M, Karavas A, Fox J, Byrne JG, Aranki SF, Couper GS (2003). Minimally invasive mitral valve repair suggests earlier operations for mitral valve disease. J Thorac Cardiovasc Surg.

[2] Sabanathan S, Smith PJ, Pradhan GN, Hashimi H, Eng JB, Mearns AJ (1988). Continuous intercostal nerve block for pain relief after thoracotomy. Ann Thorac Surg.

[3] Sabanathan S, Mearns AJ, Bickford Smith PJ, Eng J, Berrisford RG, Bibby SR, Majid MR (1990). Efficacy of continuous extrapleural intercostal nerve block on post-thoracotomy pain and pulmonary mechanics. Br J Surg.

[4] Cook TM, Riley RH (1997). Analgesia following thoracotomy: a survey of Australian practice. Anaesth Intensive Care.

[5] Rodriguez-Aldrete D, Candiotti KA, Janakiraman R, Rodriguez-Blanco YF (2016). Trends and new evidence in the management of acute and chronic post-thoracotomy pain-an overview of the literature from 2005 to 2015. J Cardiothorac Vasc Anesth.

[6] Okitsu K, Iritakenishi T, Iwasaki M, Imada T, Fujino Y (2017). Risk of hematoma in patients with a bleeding risk undergoing cardiovascular surgery with a paravertebral catheter. J Cardiothorac Vasc Anesth.

[7] Leyva FM, Mendiola WE, Bonilla AJ, Cubillos J, Moreno DA, Chin KJ (2018). Continuous erector spinae plane (ESP) block for postoperative analgesia after minimally invasive mitral valve surgery. J Cardiothorac Vasc Anesth.

[8] Chen N, Qiao Q, Chen R, Xu Q, Zhang Y, Tian Y (2020). The effect of ultrasound-guided intercostal nerve block, single-injection erector spinae plane block and multiple-injection paravertebral block on postoperative analgesia in thoracoscopic surgery: a randomized, double-blinded, clinical trial. J Clin Anesth.

[9] Yang HM, Choi YJ, Kwon HJ, Cho TH, Kim SH, O J (2018). Comparison of injectate spread and nerve involvement between retrolaminar and erector spinae plane blocks in the thoracic region: a cadaveric study. Anaesthesia..

[10] Onishi E, Toda N, Kameyama Y, Yamauchi M (2019). Comparison of clinical efficacy and anatomical investigation between retrolaminar block and erector spinae plane block. Biomed Res Int.

